# Developing assistive technology to support reminiscence therapy: a user-centered study

**DOI:** 10.3389/fmed.2025.1625897

**Published:** 2025-10-02

**Authors:** Soraia M. Alarcão, André Santana, Bruna Vieites, Pedro Neves, Raúl Alves, Carolina Maruta, Manuel J. Fonseca

**Affiliations:** ^1^LASIGE, Faculdade de Ciências, Universidade de Lisboa, Lisboa, Portugal; ^2^Laboratório de Estudos de Linguagem, Centro de Estudos Egas Moniz, Faculdade de Medicina, Universidade de Lisboa, Lisboa, Portugal

**Keywords:** assistive technology, dementia, human-centered computing, reminiscence therapy, user-centered design

## Abstract

Caregivers are one of the main pillars in adopting digital technologies for reminiscence therapy, as they are responsible for its administration. Despite their comprehensive understanding of the needs and difficulties associated with the therapy, their perspective has not been fully considered in existing technological solutions. To mitigate this gap, we followed a user-centered design approach, using a sequential process that included worldwide web-based surveys, follow-up interviews, and focus groups to inform the development of novel assistive technological solutions focused on the real needs of caregivers. A total of 713 informal and 67 formal caregivers participated in our study. Our findings reveal that caregivers are overburdened with the amount of work they have daily; thus, mechanisms that could help them manage all the tasks involved in therapy (e.g., creating sessions, gathering feedback for further consultation) would help to reduce their workload while potentially improving the quality of therapy sessions. Caregivers also want to be able to easily leverage emotions during therapy to personalize and diversify sessions over time, thus preventing aggression or agitation in people with dementia. As a result of our study, we propose a list of functional requirements gathered for both formal and informal caregivers and the corresponding expected primary and secondary outcomes, such as improvement of the cognitive function of people with dementia, reduction of caregivers' stress and burden, and reduction of behavioral symptoms of people with dementia. We also present the resulting architecture of the *KeepsakeBox* prototype, which allows creating, managing, and delivering personalized reminiscence therapy sessions to people with dementia, which can be used as a basis for the development of future technological solutions for reminiscence therapy.

## 1 Introduction

Dementia is the seventh leading cause of death among all diseases and a significant cause of disability and dependency among older people (1). There are currently more than 55 million people living with dementia worldwide, and around 10 million new cases every year. It is a clinical syndrome characterized by a significant cognitive decline in several cognitive and behavioral domains. It is severe enough to interfere with personal, occupational, and social activities (such as attention, executive function, learning and memory, and language) (2, 3). Dementia has physical, psychological, social, and economic impacts, not only for people with dementia but also for their caregivers, families, and society in general (4).

The need to improve the quality of life, independence, and engagement in meaningful activities for people with dementia, while reducing their families' burden, has become a challenge in this field, which motivated the development of intervention programs with positive outcomes (5). Reminiscence is an inexpensive non-pharmacological therapy commonly used due to its therapeutic value for elderly adults (6–9). This therapy is useful for creating engaging communication between people with dementia and the rest of the world by using the preserved abilities of long-term memory to alleviate the experience of failure and social isolation, rather than emphasizing the existing impairments. Reminiscence therapy reduces the neuropsychiatric symptoms of people with dementia, but is highly time-consuming and dependent on human interaction since it requires the acquisition of different multimedia materials to be used (5, 10–13).

Several computerized systems have been developed to help people with dementia perform everyday activities, improve the treatment of dementia by stimulating memories, communication, and social engagement, or diagnose and evaluate the progression of the disease (11, 12, 14). Current assistive technological solutions improve reminiscence therapy by providing a more positive and engaging experience to all participants (people with dementia, family members, and clinicians) while using multimedia technology to gather and store different multimedia to save them valuable time (13). However, there are mixed results regarding the benefits of assistive technology for reminiscence therapy (15) and challenges in its adoption due to high cost, lack of training and support to use the device/technology, and a lack of fit to the care environment (16). Their adoption in everyday life is still limited, but some barriers could be overcome if caregivers were consulted during the design phase (16–18).

To the best of our knowledge, and based on our review of the literature, most research has focused on people with dementia, since they are the target of the therapy, while informal caregivers have been seen mainly as aids for preparing, delivering, and evaluating sessions, which may entail an overload of work and stress for them. In fact, despite informal caregivers' comprehensive understanding of the needs and difficulties associated with reminiscence therapy, as well as being the ones making the decision on purchase, maintaining, and even abandoning the use of assistive technologies, their perspective has yet to be fully considered (18–20). To inform the design of novel assistive technological solutions focused on the real needs of caregivers, particularly informal ones, we followed a user-centered design approach through worldwide web-based surveys, follow-up interviews, and focus groups.

The main aspects covered in this study were: (i) how the socio-demographic characteristics of the dyad person with dementia and caregiver influence the realization of reminiscence therapy sessions; (ii) what are the emotional reactions and behaviors that people with dementia have during therapy sessions (in particular, the negative ones), and what are the informal caregivers' attitudes to cope with such negative emotional reactions and behaviors; (iii) what information is useful to use and collect to improve reminiscence therapy sessions over time, particularly what topics are most beneficial to engage people with dementia during therapy sessions. With this study, we have two goals. First, we aim to understand how reminiscence therapy is usually conducted and what are the needs and difficulties felt by the caregivers. Second, leveraging the knowledge gathered, we aim to investigate how novel assistive technological solutions could aid caregivers in managing and delivering reminiscence therapy sessions. This not only bridges gaps between technical development and real-world applicability but also enhances the likelihood of sustained engagement of assistive technologies in dementia care, in particular for reminiscence therapy.

## 2 Background and related work

In this section, we present some background regarding dementia and reminiscence therapy. We further outline the state-of-the-art of assistive solutions to support reminiscence therapy in dementia care.

### 2.1 Dementia

Dementia is characterized by a significant decline in several cognitive, functional, and behavioral domains. Cognitive impairments in dementia affect comprehension, language, memory, orientation, thinking, emotional control, social behavior, and motivation (8). People with dementia moving through the phases of the disease become increasingly depressed, insecure, and isolated (21). Behavioral dysfunction might come from inactivity, discomfort, and the need for social contact, but people with dementia usually spend part of their time without any engaging activity.

Over the years, the most common treatment was pharmacological medication focused only on symptomatic control of the disease (i.e., stabilization of cognitive status and reduction of behavioral changes) (7). However, in addition to its limited effectiveness, its symptomatic effects affect only a small portion of people with dementia. Non-pharmacological treatments are available, including memory training, support groups, reminiscence therapy and life review, psychodynamic approaches, reality orientation, and cognitive and behavioral therapy (7). Each may have different goals: activate memories, strengthen intact abilities, alleviate distress, facilitate coping, or enhance behaviors. They aim to improve the well-being of people with dementia and reduce caregivers' burden (9).

### 2.2 Reminiscence therapy

Reminiscence therapy is widely used in the treatment of dementia due to its therapeutic value for older adults, being a cost-effective non-pharmacological therapy (6–9). It is based on the premise that long-term memory remains intact until the latter stages of the disease. By triggering the older memories of people with dementia using specific stimuli (e.g., photos, music, videos), it is possible to create interesting and engaging conversations between people with dementia and the rest of the world. These conversations take advantage of the preserved abilities of the person with dementia rather than emphasizing the existing impairments, thus helping to alleviate the experience of failure that gradually inhabits most of their interactions (22). Joint approaches that include caregivers in the therapy improve the relationship between the caregivers and the people with dementia, benefiting both (6).

Evidence suggests that reminiscence therapy is a highly effective treatment for depression since it enhances the self-esteem and life satisfaction of people with dementia while improving their self-integrity (9, 23). The process of reminiscence is associated with providing pleasure, security, health, and a feeling of belonging to a place (22). It also presents slight interpersonal (enhancing the relationship between the caregiver and the people with dementia, and overall socialization) and intrapersonal (self-awareness, personal stability, and life meaning) benefits (7). Interpersonal benefits are more useful for individuals within a moderate stage, while intrapersonal ones are more useful for the mild dementia stage.

### 2.3 Assistive technology for reminiscence therapy

Digital technology has become an indispensable tool that can be used to promote independence, positive moods, and behavior of people with dementia (5, 24). Over the years, a growing body of research has been presented to help people with dementia perform everyday activities, improve the treatment of dementia to stimulate memories, communication, and social engagement, and diagnose and evaluate the progression of the disease.

#### 2.3.1 Multimedia systems

The use of multimedia to support reminiscence has been a topic of interest over the years to improve memory recall, promote social interaction, and support personalized care. CIRCA, CART, Companion, InspireD, among many others, aim to support memory recall and improve communication, engagement, and autonomy using videos, music, and virtual environments (22, 25–30), provide personalized, positive, and stimulating experiences using multimedia and trusted messages (11, 24, 31–34), as well as support psychological stability and well-being by combining photo sharing with a schedule prompter system for daily tasks (35, 36).

Other initiatives, including Collegamenti, MyStory, and Portrait, have focused on supporting meaningful interactions and collecting stories that summarize important moments in the life of people with dementia to facilitate reminiscence therapy, for example, to assist care staff to learn more about residents' personal histories (37–43). LifeBio Memory leverages speech-to-text to minimize the need to hand-write or type individualized life story data (44), and Recuerdame assists occupational therapists in planning, organizing, and documenting reminiscence therapies for people with dementia (45). Pensieve supports people's reminiscing practices by reusing memory-rich content from social media, proving to be a meaningful and effective approach, although not specifically targeting people with dementia (46, 47).

#### 2.3.2 Games and VR/AR interventions

Besides using interactive multimedia to support reminiscence therapy, authors have explored using games and Virtual Reality (VR)/Augmented Reality (AR) to create immersive experiences. Different types of games, in particular serious games and exergames, have been used to promote autonomy (48), memory recall (49), as well as sustained interaction and engagement (50–52). For example, Memory Matters uses interactive activities to tap their long-term memories using games that could be played solo or in groups (49), while SAUDÆDE focuses on personalizing the game content with musical elements, photos, and biographical details (52).

VR/AR solutions used highly realistic, real-time rendered 3D environments with gesture-based technology to navigate through space and time, and Head-Mounted Displays (HMDs) to deliver tailored reminiscence therapy for people with dementia. The proposed solutions have achieved promising results in eliciting memories (53–55), fostering social interaction (between the staff and residents with dementia) (56, 57), enhancing the feeling of involvement/presence (56, 58), promoting emotional well-being (54, 59–63), reducing behavioral symptoms (57, 59–63), and improving verbal fluency (64). Moreover, Lau et al. propose a novel conceptual model centered around immersion, interaction, imagination, and impression, providing insights for future development of VR to support reminiscence therapy (65).

#### 2.3.3 Tangible objects and multisensory interfaces

More recently, tangible objects and multisensory interfaces have been used to enable spontaneous reminiscence therapy (66–68), facilitate intergenerational communication, reminiscence, and social engagement (69–71), increase people with dementia's autonomy, independence, and quality of life by allowing them to do the reminiscence activity by themselves (72), enhance the sense of familiarity and comfort (73), improve communication and emotions (66). Authors resorted to wearable technology linked to nearby artifacts (e.g., pot with herbs) (67), tangible multimedia books combined with audio (69, 70), a chest of drawers where each one contains items representing sub-topics (using images) (72), wall-mounted interactive tool depicting an old-style radio/TV (73), an interactive picture frame with various sensory outputs (video, sound, objects, and smell) (66), and a multisensory, themed table-top boxes, combining physical and digital elements (71).

#### 2.3.4 Conversational agents, intelligent virtual agents, and social robots

Recently, there has been an increase in the number of works that leverage conversational and intelligent virtual agents as well as social robots to support reminiscence therapy. Conversational agents have been used to help people with dementia reminisce (74–78), provide engaging and challenging sessions (75, 79), improve emotional context understanding and boost conversation engagement (79), and enable meaningful conversations (80). For example, ReminX uses a custom AI chatbot to interact with family members to gather short audio notes and photos to create rich documentary-like stories (74). Elisabot simulates a reminiscence therapist by asking questions about the patient's experiences using photos to generate life-related questions, but it requires human support (75). The Know Me Inside-Out collects and structures patient memories into context-aware quizzes using named-entity recognition, question generation, and sentiment analysis (81). The AMPER application used an intelligent virtual agent to convey friendliness, kindness, knowledgeability, and trustworthiness to ensure the agent's effectiveness in supporting therapy sessions (77).

MARIO companion robot aims to support people with dementia, their caregivers, and healthcare professionals by delivering personalized reminiscence therapy (through familiar content like music or books) and autonomously conducting comprehensive geriatric assessments (82, 83). Yuan et al. leverage reinforcement learning to adjust the social robot conversation strategies to provide memory triggers and tailor prompts to maintain a positive emotional state for people with dementia during reminiscence therapy (84). Finally, the social robot Pepper uses adaptive AI to automatically display personalized memory triggers (like photos and videos) to engage people with dementia in conversations during reminiscence therapy (85). The robot's ability to adjust its body language and voice facilitates both verbal and nonverbal interactions.

#### 2.3.5 End-user involvement in ATs for reminiscence therapy

When designing any technological solution, it is essential to understand the needs of the end-users of such solutions, in this case, not only people with dementia, but also their caregivers, who work as their proxies and execute the reminiscence therapy sessions. End-user involvement is typically implemented through participatory design and user-centered design. In participatory design, users are seen as active collaborators helping to co-design the technology, while in user-centered design, the focus is on understanding users' needs and considering their feedback throughout the design process, without necessarily involving them as co-designers.

Although we believe that the authors of the various solutions have implicitly attempted to take into account the needs of the various users (people with dementia, informal caregivers, and formal caregivers), in most instances, this is not clearly stated. Requirements gathering (before the development of the solutions) was only performed on about 30% of the existing solutions. Ly et al. (72), Ibarra et al. (40), and Unbehaun et al. (50, 51) involved only people with dementia (average of 41 participants). Cohene et al. (11, 31) and Piper et al. (69) involved one person with dementia each alongside their relatives. Sarne-Fleischmann et al. (27, 28), Siriaraya et al. (56), Edmeads et al. (42), Paay et al. (67), I-Jui2024 et al. (55), and Matsangidou et al. (61–63) involved on average 16 people with dementia and 22 formal caregivers. Finally, Gowans et al. (25, 26), Aylett et al. (77), De-Rosende-Celeiro et al. (45), Gerbaudo-Gonzalez et al. (30), and Xygkou et al. (80) involved, on average, five formal caregivers. It is worth noting that several authors did not disclose detailed information on the number of either people with dementia or formal caregivers involved in the requirements phase (25–28, 40, 42, 55, 67). Overall, when different stakeholders participate in the requirements phase, it usually involves a small number of people with dementia and formal caregivers in partnership with local institutions, leaving informal caregivers excluded from this process.

The effort the authors of most solutions made in ensuring that they were evaluated by people with dementia or dyads caregiver-people with dementia is noteworthy. Around half of the technological solutions analyzed evaluated their impact on people with dementia or dyads (on average 15±17 participants), mostly at institutional settings. Of particular note were the evaluations made on the solutions presented in Yasuda et al. (37) with 15 participants, Saredakis et al. (64) with 17 participants, Siriaraya and Ang (56) and Matsangidou et al. (61–63) with 20 participants each, Gowans et al. (25, 26) with 21 participants, Casey et al. (82) with 22 participants, Laird et al. (33) with 30 participants, Tominari et al. (54) with 52 participants, and Yu et al. (49) with 80 participants.

Technological solutions to support reminiscence therapy have advanced from static digital memory aids to immersive, adaptive, and AI-powered systems toward a more personalized and engaging experience. However, early-stage involvement in the design process featured small groups of formal caregivers or people with dementia, with many studies lacking detailed participant data. Overall, user participation in the design process remains limited. As stated by Edmeads et al., this may lead to potentially inaccurate assumptions made about the issues and needs of people with dementia and caregivers, and as a result, about the technology that may be appropriate to address them (42). Consequently, it might compromise the adoption and suitability of such technology for the users. This is particularly relevant for home-based reminiscence therapy with informal caregivers, who are still not considered when developing technological solutions despite being the ones using them (16–20).

## 3 User-centered design study to identify caregivers' needs

Our main goal with this work is to inform the design of novel assistive solutions within dementia care, particularly focusing on reminiscence therapy. For that, it is crucial to include caregivers in the loop to identify their needs, ensuring they are reflected in the functional requirements of the assistive solutions to be developed. Following, we describe our study's method and the results achieved.

### 3.1 Method

**Figure 1:**
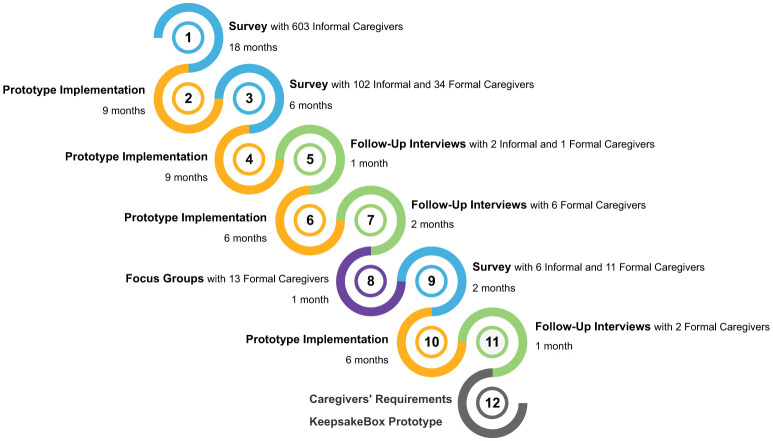
User-centered design followed (figure best seen in color).

We followed a user-centered design (86) in which the results of each step of the study inform the next ones (see Figure 1). We combined three surveys with interviews and focus groups to progressively deepen our understanding of the needs of people with dementia caregivers in the context of reminiscence therapy. A total of 713 informal and 67 formal caregivers worldwide participated in our user-centered design study.

Given that the state of the art was focused mostly on formal caregivers and institutional settings, we started with a survey targeting informal caregivers to identify the needs of people with dementia and their informal caregivers when performing reminiscence therapy (step 1). Our first survey highlighted the main challenges faced by informal caregivers during reminiscence therapy, which directly shaped the design of our second survey. This entailed the inclusion of formal caregivers and validation items to cross-check whether formal caregivers observe similar challenges that informal caregivers reported, and how to cope with them. This decision arose after many informal caregivers in the first survey reported feeling unsupported, overwhelmed by the amount of work they have, and unsure of how to deal with people with dementia during emotional outbursts.

Our second survey was aimed at both informal caregivers to validate findings from the previous survey and formal caregivers to also identify their needs in the context of therapy performed in health institutions (step 3). Our findings from the second survey confirm some of our previous findings but also revealed divergent needs between informal and formal caregivers, prompting a focus toward practical implementation aspects. Such aspects were then addressed in the third survey, particularly regarding data capture and visualization, i.e., to identify what information should be collected from sessions and how caregivers prefer to visualize it (step 9).

Follow-up interviews (steps 5 and 7) and focus groups (step 8) were conducted to gather more in-depth information and validate our main findings from the surveys. At each step of the user-centered design followed, the *KeepsakeBox* prototype^1^ was evaluated with the caregivers, and improved based on the suggestions made (steps 2, 4, 6, and 10). The developed prototype fulfills two roles in our work. Firstly, it was used as a tool to elicit and validate the functional requirements. Secondly, the resulting architecture is offered as a backbone for future assistive technological solutions. As a result of the study conducted, we present a set of validated functional requirements gathered for both formal and informal caregivers, and the resulting architecture of the *KeepsakeBox* prototype developed during the requirements elicitation process.

Following, we detail how the participants were selected, the materials used, and the procedures adopted for the data collection and analysis of the results.

#### 3.1.1 Participant selection

Recruitment occurred via social and institutional networking, and caregivers' participation was voluntary. The main inclusion criteria were being (or having been) a formal or informal caregiver of people with dementia. We did not intentionally impose additional inclusion or exclusion criteria, such as age, years of experience, or care setting. This decision was driven by our user-centered design approach, in which we sought to identify a wide range of caregivers' experiences and perspectives to inform the development of novel assistive technologies. Applying a more restrictive set of criteria might have limited the diversity and ecological validity of the insights obtained.

Both the surveys and the interviews/focus groups invitations were disseminated in Facebook^2^ groups selected according to the following keywords: *alzheimer, demência, demens, demenz, demenza, dementia, huntington, parkinson, pick*, and *vascular dementia*. We contacted several social/community/healthcare institutions to disseminate the surveys through their communication channels. The process of selecting institutions was similar to the one used to select the Facebook groups.

We used a purposive sampling strategy to select participants for prototype validation, i.e., caregivers who volunteered to participate in the interviews during the surveys were contacted and invited to participate, as well as additional caregivers identified through our collaborating institutions or suggested by caregivers who had already participated. This selection aimed to ensure representation from both formal and informal caregivers with varying experience levels and familiarity with technology. However, the anonymity of responses prevents confirmation of whether there are recurring participants between the different phases of the project.

#### 3.1.2 Materials

The first survey consisted of 65 open and closed-type questions tackling four domains: socio-demographic characteristics (of both informal caregivers and people with dementia), reminiscence therapy, the use of an Life Story Book (LSB), and interest in technological features. Our second survey had two versions: one focused on informal caregivers, and another on the formal ones. The version for the informal caregivers consisted of 38 open/closed questions, while the one for the formal caregivers had 36 open/closed questions. Both versions tackled the same domains: socio-demographic characteristics of the caregivers, people with dementia follow-ups (reminiscence sessions, follow-up appointments, and other forms of follow-up), and interest in technological features. Our third, and final survey, consisted of 44 open and closed-type questions focusing on five domains: socio-demographic characteristics of the caregivers, logging information about sessions, session visualization, information about the sessions to be displayed, and visualization of statistics from the sessions. Closed-type questions included Likert-type scales, multiple choice, and dichotomic items.

Interviews and focus groups focused on the caregivers' characterization, their understanding of what reminiscence therapy is, the care they provide, and which technology they are familiar with. Our aim was to attain more in-depth information regarding the following topics: (i) identify which materials caregivers used, how they acquire such materials, and the role of personalization in reminiscence sessions; (ii) identify mechanisms to help in the creation of sessions; (iii) understand which information is useful to be registered from the sessions performed and how it should be visualized for further consultation; finally, (iv) how to support the communication and share of people with dementia care among caregivers.

#### 3.1.3 Procedures

Our study was designed in accordance with the Declaration of Helsinki and national and legal regulatory requirements. There were no anticipated risks involved in the participation. The study protocol was submitted to the local Institutional Review Board and was exempted from the full ethical application.

Surveys, interviews, and focus groups started with an introduction of the aim of our study, an explanation of the informed consent procedure, and information on the confidentiality and anonymity of the research data. Participation in each phase of the study was voluntary, and participants could withdraw at any point. Moreover, they were not paid money nor received any other type of compensation, and they all provided their consent. We made our surveys available through a link to the Google Forms platform.^3^

We conducted the semi-structured interviews and focus groups remotely using the Zoom platform,^4^ following a think-aloud protocol (87) during the demonstrations of the *KeepsakeBox* prototype led by both the first and last authors, with the assistance of the remaining ones. Participants were asked to reflect on the themes identified in the surveys, provide elaboration, and offer feedback on preliminary design directions, such as the types of media and topics to include in reminiscence sessions, preferences for how session data should be visualized, and which information should be collected. Participants were also encouraged to verbalize their thoughts, feelings, and decision-making processes while interacting with the prototype, as well as assess the usability of the proposed interface and interest in the developed features. These qualitative sessions validated survey findings and uncovered subtleties and situational nuances that surveys alone may not detect. This method allows us to identify usability challenges and users' needs for refining/validating the system requirements and outcomes.

Data collection was anonymous, and no personally identifiable information was collected, except for the e-mail (provided by the subject if they wished to be informed about our future work), which was only accessible to the investigators and kept confidential and off the records. The e-mail was collected on a separate form not linked to the survey, guaranteeing the anonymity of the participants' answers. Incomplete or duplicate questionnaires were excluded.

#### 3.1.4 Data analysis

We conducted a mixed deductive and inductive thematic analysis on all the open-ended questions of the surveys (88). The content of open questions was analyzed several times and further categorized (in a coding frame). Two independent investigators coded each response according to the coding frame developed. This coding frame was used as a basis for the second survey, adapted according to the survey's characteristics. A new coding frame was developed for the third survey using the same methodology. For all the surveys, two other investigators acted as independent referees in case of divergence.

Descriptive statistics summarized demographic variables and categories. Since all of our data involved nominal or ordinal variables, all statistical tests conducted were non-parametric: Spearman Rank, Chi-Square/Fisher's exact test, Kruskal–Wallis *H*, and Mann–Whitney *U*. If the Chi-Square/Fisher's exact test was significant, adjusted residuals with Bonferroni correction *post hoc* analysis were conducted. If the Mann–Whitney *U* test was significant, Kruskal–Wallis *W* was used as a *post hoc* analysis. Finally, if Kruskal–Wallis *W* was significant, a pairwise *post-hoc* Dunn test with Bonferroni adjustments was conducted. Effect sizes were calculated using Cramer's *V* and Glass's Rank-Biserial Correlation. Results of statistical tests were considered significant if the *p*-value was below 0.05. Analyses were performed using IBM SPSS Statistics. Interviews and focus groups were recorded (with participants' permission), and their audio recordings were transcribed for analysis. We used verbatim transcription since it accounts for a richer interaction experience, thus being more appropriate for qualitative research (89). Two independent investigators coded each interview and focus group, and a third investigator acted as an independent referee in case of divergence.

### 3.2 Results

Next, we summarize the results obtained from all steps of our study (recall Figure 1).

#### 3.2.1 Participants

A total of 713 informal and 67 formal caregivers worldwide participated in our study. Ninety-two percent of the informal caregivers are female, older than 50 years old (58%), completed higher education (69%), have mild to moderate visual impairments (56%), and are either the children (59%) or the spouse of the people with dementia (24%). They find themselves in distress and overburdened with their daily work while caring for the person with dementia.

Eighty-four percent of the formal caregivers are female, younger than 50 years old (64%), and have completed higher education (89%). They are mainly psychologists, neuropsychologists, recreation therapists, nurses, and speech-language pathologists. On average, they take care of more than 38 people with dementia (varying from 4 to 197).

Most caregivers are familiar with recent technology. Informal caregivers use smartphones 5–6 days a week and computers/tablets/iPads 3–4 days a week. Formal caregivers use smartphones daily and computers/tablets/iPads 5–6 days a week.

#### 3.2.2 Socio-demographic characteristics' influence on the execution of reminiscence therapy

Informal caregivers reported that 65% of people with dementia were performing reminiscence therapy at the time of the surveys, while 23% stopped doing it due to increased detachment of the person with dementia (66%), lack of perceived usefulness or inability from the person with dementia to attend sessions (25% each), lack of material (10%), and lack of time (7%). The remaining ones (12%) never performed reminiscence therapy, as it was prevented by a detachment of the person with dementia (66%), lack of perceived usefulness or the person with dementia not being able to participate (25% each), lack of time (8%), and lack of materials (7%).

We analyzed whether performing reminiscence therapy was associated with the overall daily stress, as well as the daily stress and daily work of informal caregivers, specifically due to the care of the person with dementia, and the socio-demographic characteristics of the informal caregivers and people with dementia. There were no significant relationships for all variables except for the disease's stage and the informal caregiver's daily work.

There was a statistically significant moderate relation between performing therapy and the stage of the disease [χ^2^(6) = 54.461, *p* < .00001]. *Post-hoc* analysis suggests that people with dementia in the initial stages of the disease perform therapy more often: 81% of those at a mild stage and 74.9% at a moderate stage perform therapy, against only 47.1% at a severe stage. As the disease progresses, there is an increase in the number of people with dementia who stop performing therapy: 37.1% of those at a severe stage and 16.3% at a moderate stage stopped performing therapy. In contrast, only 7.1% of those at a mild stage stopped.

The daily amount of work of informal caregivers due to caring for the person with dementia is statistically significantly different across those who perform (or do not) the reminiscence therapy [χ^2^(8) = 19.409, *p* = 0.013]. *Post-hoc* analysis revealed, as one might expect, that when the amount of daily work with the person with dementia is higher, therapy is performed less often.

#### 3.2.3 People with dementia's emotional reactions during reminiscence therapy sessions

When reviewing memories, it is common that each person has different emotional reactions depending on the memory in question. During sessions, informal caregivers reported that they either perceive what the person with dementia is feeling (62%) or the person with dementia can describe what they are feeling (46%). Sensorial and emotional fluctuations as well as difficulties with emotional regulation frequently occur throughout the sessions (64%), resulting in negative emotional reactions and behaviors. Caregivers report that people with dementia become agitated, aggressive, detached, and anxious (56%), they try to talk about what is happening (49%), and get emotional, mainly through crying (47%). The main situations in which people with dementia experience such negative emotions are related to the existing cognitive impairments (58%) and their autobiographical memories (44%). Examples of cognitive impairments or autobiographical memories that trigger negative emotional reactions in people with dementia are:

- **Not being able to remember something:**

“*When memories don't line up with reality.”* [I-55]

- **Becoming confused:**

“*When she gets confused with names relating to a photo, but can remember why it was taken.”* [I-164]

- **Inability to express what they are feeling:**

“*When she can't find words to describe her memories, she gets very frustrated, angry.”* [I-33]

- **Remembering (or seeing pictures of) loved ones who passed away:**

“*Gets sad when seeing photos of his deceased wife and sister.”* [I-23]

- **Bad memories from their lives:**

“*Remembering the father hitting his mother.”* [I-369]

#### 3.2.4 Caregivers' attitudes toward people with dementia's emotional reactions

Formal caregivers are skilled in addressing the negative emotional reactions and behaviors that occur and in leveraging them for therapy. Thus, we focused on figuring out how informal caregivers cope with such situations, given that they usually have the most difficulties doing so. To cope with the negative emotional reactions and behaviors that occur, informal caregivers adopt different strategies: verbal actions toward the person with dementia (67%), changing the environment, theme, or activity (50%), and physical actions toward the person with dementia (21%).

Informal caregivers usually do a mix of talking and touching/hugging the person with dementia, trying to calm them down by rationalizing and validating what the person with dementia is feeling, as well as helping them to deal with the triggered memories (e.g., helping them to remember the situation or a particular person in a photo). However, as we anticipated, when people with dementia become agitated, aggressive, or angry, it is not uncommon that informal caregivers do not know how to deal with the person with dementia properly, nor how to help them:

“*I yell because I get so frustrated.”* [I-148]

“*Sometimes I don't know what to do.”* [I-534].

Ultimately, these situations impair not only the emotional state and quality of life of people with dementia and their caregivers but also the quality of their relationships.

#### 3.2.5 Topics to engage people with dementia during reminiscence therapy sessions

Multimedia materials play an important role in reminiscence therapy. Different multimedia could be used during each session, such as images, music, video, text, or combinations of these. The main goal is to use materials that can attain an overall positive and stimulating experience to engage the person with dementia while helping them in the process of reminiscing. In Table 1, we provide a comparison of the multimedia used by informal and formal caregivers.

**Table 1:** Comparison of the multimedia used by informal and formal caregivers.

	**Informal caregivers**	**Formal caregivers**
Types of media used	- Images (60%)	- Images (95%)
Personal vs. generic material	- Personal material (89%)	- Personal material (87%)
Common topics in materials	- Relatives (93%)	- Places lived (96%)
Sources of material	- Relatives (81%)	- Internet (87%)
Update materials' frequency	- Daily (29%)	- Monthly (26%)
Barriers to updating materials	- Detachment of the person with dementia (50%)	- Lack of time (100%)

Both formal and informal caregivers use multimedia materials, with a strong preference for images and music in both groups. However, formal caregivers tend to use a wider variety of formats, including videos and texts, more frequently than informal caregivers. Both groups use personalized materials related to the person with dementia since personalized multimedia is a better memory trigger than generic multimedia because it is part of that person's life. However, as pointed out by caregivers during the interviews and focus groups, the use of both is valuable and desirable:

“*Using images and music from when they were in their 20s mostly and family images so can reinforce connections. Also, podcasts of former interests mum likes to do: peeling vegetables, folding...”* [I-712]

“*I often start by showing generic images and songs, and then I'll try to narrow it down a little bit. If I know a person likes some particular ones, or it makes a greater impact on them, I make it more personal.”* [F-46].

The topics covered in the materials used during sessions are broadly similar between formal and informal caregivers: family members, places where the person has lived or visited, and previous jobs. However, formal caregivers report that they would like to use material related to other topics such as habits, hobbies, favorite food, musicians, television shows, movies and actors, important dates, past and present objects, sports, or plants. Informal caregivers often obtained such materials from family members, while formal caregivers more frequently relied on the internet, which may reflect different levels of access and familiarity with the person's life history. Finally, both groups more or less regularly update the materials, but informal caregivers are more likely not to update them at all, possibly due to stress or lack of time. Although lack of time is a common barrier, formal caregivers also mention practical limitations, such as the need to repeat content or doubts about the effectiveness of the material.

Overall, existing differences reflect the distinct care contexts in which reminiscence therapy is conducted, i.e., family and home vs. professional and institutional, that influence access to resources, availability of time, and perceptions about the impact of multimedia materials.

#### 3.2.6 Information to collect to improve reminiscence therapy sessions over time

Collecting feedback from the sessions performed is very important since it allows caregivers to compare sessions and customize them over time to deliver better therapy, tailored to the current condition of each person with dementia. Table 2 presents a comparison of the information collected by informal and formal caregivers.

**Table 2:** Comparison of the information collected by informal and formal caregivers.

	**Informal caregivers**	**Formal caregivers**
Emotional reactions	- Positive (57%)	- Positive (83%)
Memory recall	- Yes (55%)	- Yes (83%)
Emotional state	- Before (38%)	- Before (65%)
(before/after session)	- After (38%)	- After (87%)
Session duration	- No	- Yes (48%)
Engagement and participation	- No	- Record the level of involvement, interest, and communication of the person with dementia
Relational dynamics	- No	- Conduct periodic cognitive assessments
Emotional state	- No	- Before (65%)

Both informal and formal caregivers monitor which multimedia materials elicit positive emotions or help recall memories, and both frequently monitor the emotional state of the person with dementia before and after sessions. Formal caregivers take a more detailed approach, also recording session duration, engagement, communication, and conducting cognitive assessments. They reported being interested in registering the intensity of emotional reactions, behavioral cues, session refusals, and other factors that could inform future care strategies. These differences are likely due to the different care settings, i.e., informal caregivers conduct the sessions at home, with limited time and training, while formal caregivers work in professional settings, where comprehensive documentation facilitates therapeutic goals.

During the interviews and focus groups, several suggestions were made and discussed regarding which information should be collected (and how). The general consensus is that the process should be as simple as possible. It should be possible to review the multimedia content shown during the session, and for each one, collect the emotional reactions of the person with dementia (negative, neutral, and positive). Further granularity in the information collected is only helpful in the case of negative emotional reactions. Ways to visualize the evolution of the person with dementia over time are also desired. It was also pointed out that the caregiver should only need to input a small amount of feedback for each session, as it can be bothersome on a daily basis. To avoid upsetting the person who is undergoing the therapy, feedback should, ideally, be collected at the end of the session or after consulting the session summary (even days later):

“*It also makes sense to have a summary that serves as a profile of the work I have been doing with the person with dementia, and that guides me later in the intervention: when did I start, when did I have last session, how many sessions have I done, what themes have I approached, what themes have I approached that were more successful, what should I not present again as a stimulus (to the person with dementia).”* [F-9]

“*We are always following the person very closely, moment to moment, and so we leave a little bit of that part (of recording the information) for later. So that the person doesn't feel that ‘Oh, he seems to care more about recording than being with me and talking to me'. People, when they are with us, need to feel that we are ‘whole' in what we are doing with them..”* [F-46]

#### 3.2.7 Caregivers' interest in technological functionalities to support reminiscence therapy

We surveyed caregivers about their interest in technological functionalities that could aid them in managing, delivering, and adapting the reminiscence therapy sessions over time. Informal caregivers are moderately interested in all the technological functionalities (median of 4 in 5 for each) that may help to revisit older memories of their loved ones. Formal caregivers are also interested in all the technological functionalities. In particular, they are very interested in those related to the adaptation and personalization of sessions (median of 5 in 5 for each). Compared to informal caregivers, their interest is much higher, which we believe is because most informal caregivers only take care of one person and do not need to manage several people with dementia simultaneously. Such a difference in interest across the type of caregivers is statistically significant for all the functionalities under evaluation (see Table 3).

**Table 3:** Comparison between informal and formal caregivers regarding their interest in technological features.

	**Technological functionality**	***U***	***p*-value**	***r* (effect size)**
	Creating reminders	1,063.50	< 0.001	–0.387 (medium)
Management of people with dementia	Notifying other caregivers	1,089.00	< 0.001	–0.372 (medium)
	Automatically adapt the session content based on the biographical information	758.50	< 0.00001	–0.563 (large)
Reminiscence therapy sessions	Consulting the multimedia materials used in each session	801.00	< 0.00001	–0.538 (large)
	Inserting new material	862.50	< 0.00001	–0.503 (large)
Material used in the therapy sessions	Inserting new topics relevant to the person with dementia	891.00	< 0.001	–0.486 (large)
	Email/telephone	1,276.50	0.018	–0.264 (medium)
Help tools	Help within the application	1,168.50	0.004	–0.326 (medium)

A negative Rank Biserial Correlation (*r*) value indicates that our second group (formal caregivers) tends to have larger values on their interest level than the first group (informal). We used the effect size criteria for Pearson correlation coefficients of 0.1 = small, 0.3 = medium, and 0.5 = large to interpret the *r* value (90).

## 4 Key factors for assistive technology within dementia care

Drawing from the findings of our study, we discuss and summarize the aspects that we consider key factors when developing novel assistive technological solutions for reminiscence therapy that address the needs of caregivers. We close this section by presenting our proposed list of validated functional requirements for both formal and informal caregivers. We also present the expected primary and secondary outcomes from each requirement for people with dementia and their caregivers.

### 4.1 Supporting the creation of reminiscence therapy sessions

Caregivers are subject to high levels of stress and are overwhelmed with the amount of work they have (in particular, informal caregivers). Thus, it is crucial that novel technological solutions within dementia care, particularly those targeted at reminiscing, can easily support and deliver reminiscence therapy. To accomplish this, we suggest three complementary technological approaches to support the creation of therapy sessions in order to reduce the amount of work caregivers would have to prepare and conduct the sessions:

- **Automatic**: the caregiver would be responsible for selecting the duration and number of images to be presented in the session. The choice of images to be shown would be left to the technological solution by taking into consideration all the information available (e.g., biographical, emotional);
- **Semi-automatic**: similar to the automatic approach, but considering a list of topics selected by the caregiver. Caregivers would then have the possibility to review the session created and make changes if they want to;
- **Manual**: all details would be customized by caregivers.

We hypothesize that the first approach will benefit informal caregivers who are overburdened with the care of the person with dementia and are physically and emotionally worn out, since they would not need to prepare the session. As a result, they can perform the therapy more often and focus on the memories and communication generated. The third approach will be more useful for formal caregivers, who need more control in conducting the sessions (based on the different psychological aspects to be worked on in each one). Finally, the second approach would be helpful for both informal caregivers (those who are more proficient in technology and more willing to personalize sessions) and for formal caregivers (who want to speed up the creation of sessions without needing full control in the process). Overall, all the approaches were well received by the caregivers:

“*It makes sense (the idea of the automatic session), it even makes sense that there are already some photos available, but it should be possible for people to associate other meaningful photos with the person. It would be useful if they already had some material that would allow them to quickly do a session with that person, even if it is not highly personalized... the ideal would be to be personalized but personalized sessions are not always done according to the experiences and tastes of each person. It would be important not only for the informal caregivers but also for the technicians.”* [F-53]

Another important aspect to keep in mind when designing novel assistive solutions is to keep them relatively low-tech to ensure that informal caregivers can successfully use them at home, thus avoiding abandonment of the therapy due to difficulties with the technology (e.g., devices they are not able to operate without third parties help) or financially expensive technologies (e.g., virtual reality with multiple displays and headsets or robots). The user interface should be easy to use so that less technologically savvy caregivers can use technology to their advantage, particularly the informal ones.

The aforementioned aspects are aligned and reinforce existing literature on assistive technologies in dementia care, particularly regarding personalization, simplicity, and caregiver adaptability in designing effective technological solutions. For example, previous research has emphasized usability and perceived usefulness as important adoption indicators among informal caregivers who often lack technical training (16, 17, 91). Our three proposed session creation modes (automatic, semi-automatic, and manual) align with these ideas, offering flexibility that caters to caregivers' different needs and technical capabilities. Our emphasis on easily accessible low-tech and low-cost solutions aims to ensure that technologies are practical and adaptable to real-world care contexts, as pointed out by Hirt et al. (92) and Ienca et al. (93).

### 4.2 Personalized and diversified reminiscence therapy sessions

Despite the usefulness of existing technological solutions, most of them use the same generic multimedia data throughout the therapy sessions. Personalized multimedia is well-suited for reminiscence therapy, and it should be incorporated into technological solutions, as generic content alone may not generate the desired response from the therapy (94). Nonetheless, generic multimedia might be useful as well. For example, generic content can be used when the formal caregivers are not able to get personalized content, or to prevent a person with dementia from feeling frustrated because they cannot remember a specific event. An example of a source of personalized content is Life Story Books. They are frequently used as a reminiscence tool to support recollecting autobiographical memories (95).

Life Story Books result from a life review process performed by the person with dementia, with the support of staff and family members, to build a personal biography that acts as a bridge to the past and a connection to the present. It can be a very time-consuming task since it may require help from third parties to collect the information, which may not always be feasible or practical, as there is no simple way to do it. Embedding life story books in the technological solutions might work as a starting point to create the initial sessions and diversify and personalize the following sessions based on the biographical information of each person with dementia. Thus, their use may prevent drop-outs due to the lack of material to be used in sessions, as well as to provide valuable information to formal caregivers to stimulate the memories of the person with dementia they care for:

“*Creating a book of memories is not only useful for the person with dementia but also for family members who may not always know very much about the person and the life they led many years ago. Family history is often lost because people wait too long to ask the right questions.”* [I-123]

“*Often people in my father in laws care home haven't got information to work with.”* [I-195]

Another strategy to cope with the existing lack of personalization and diversification is to include mechanisms that effectively search for similar multimedia to diversify the pool of available images for the sessions. For example, the solutions could obtain images that are similar to the ones that a person with dementia enjoys seeing, or if the person with dementia enjoys a specific topic, new images related to that topic could be gathered as well. The acquisition of new images should be made automatically, reducing the caregiver's intervention in this process as much as possible. Such images can also be shared between people with dementia with similar interests, and even among caregivers (whenever appropriate):

“*It makes much sense (to be able to make an image public), of course. Some images are very specific to the person with dementia but can also be used with others.”* [F-50]

“*Let us imagine a caregiver feels that a particular image benefits the people with dementia they care for, a mechanism to suggest that image to caregivers of other people would be useful.”* [F-51]

Our design implications build upon and expand existing research on the customization of reminiscence therapy. Prior research has highlighted how the use of person-centered content is able to improve the engagement and emotional well-being in people with dementia, particularly when using tools like Life Story Books or personalized media collections (94, 95). Moreover, unlike previous works that often use the same content [either generic or personal (26, 38, 42, 66, 83)], our study stresses caregivers' perspectives on the difficulty of acquiring and curating both diversified and personalized materials. To bridge this gap, we identified tangible technological features, such as the automated content acquisition, public image sharing, and integration of biographical data, extending existing strategies by proposing scalable and shareable content-generation mechanisms, while challenging the current reliance on caregiver-driven customization alone.

### 4.3 Emotion-aware reminiscence therapy sessions

Reminiscence therapy sessions, ideally, should be a safe means for communication, stimulation of past memories, and, ultimately, an enjoyable and engaging experience. However, it is common to occur strong negative emotional reactions from people with dementia, which harms not only their health but also the health and well-being of their caregivers (since people with dementia tend to become aggressive or irritable). Ultimately, it will hinder therapy success and potentially harm the relationship between people with dementia and their caregivers. Despite this, existing technological solutions do not consider the emotions associated with the information collected, nor the emotional reaction of people with dementia to the presented information.

Novel assistive solutions must be able to include emotions in the loop during reminiscence therapy to facilitate the personalization and diversification of sessions over time by adapting the content being shown considering what a person is currently feeling (e.g., if strong negative emotional reactions occur), as well as by using all the biographical and emotional information available when creating new sessions. For that, such solutions should be able to either identify what the person with dementia is currently feeling (e.g., using physiological signals) or to collect such feedback from the caregivers. They should also focus on the emotions that a given multimedia can evoke in the person with dementia, which will allow them to automatically identify multimedia that will potentially trigger negative emotions in people with dementia during sessions.

Our findings further expand previous research by suggesting that emotional responses may play a critical role in the effectiveness of reminiscence therapy for people with dementia, emphasizing the need for dynamic and context-aware emotional adaptation over time. Some existing technological solutions aim to take emotions into account, but even so, they do not explore them as profoundly as would be desirable, or have some limitations. For example, in Casey et al. (82) and Asprino et al. (83), companion robots try to assess the emotional state of the person with dementia through their speech, which may not always be feasible in moderate or advanced stages of dementia, to facilitate understanding or adapt the session. Although this information is used in subsequent sessions, it is only suitable as a one-on-one therapy where the robot assumes the role of the caregiver and does not consider a scenario where a human caregiver is present. Its adoption may also be compromised by logistical and pricing issues. In contrast, our approach proposes a more flexible and caregiver-inclusive strategy where emotional responses, whether detected automatically or reported, can continuously inform and adapt session content in a simple, easy-to-use, and affordable system. This expands upon prior works by integrating emotional reactivity into both personalization and session planning processes, thereby aiming to improve therapy engagement while reducing emotional distress for both people with dementia and their informal caregivers.

### 4.4 Centralized care among caregivers

The number of people with dementia that a caregiver takes care of, although variable, can be considered large, in particular for formal caregivers. Thus, a simple centralized way to manage the information of people with dementia is a must-have. Caregivers appreciate the idea of having a profile for each person with dementia, where they can easily find relevant information about the person:

“*It should be possible to have information regarding the interests of the person with dementia, possibly previous interests, but also current interests, to have this a little bit more level-oriented. In the sense that it would be more convenient to have the information organized, and it would be easier for the various caregivers to access the information more directly.”* [F-57]

Moreover, the need to share the care of a person with dementia between caregivers is a reality for both informal caregivers (to feel more supported, less burdened, and less isolated) and formal caregivers (to optimize the relationship with other caregivers who intervene with the person, and improve the care provided):

“*This sharing doesn't have to be only with the healthcare technician, for example, our mutual daughter is not in the country. I could share, and she could remotely follow the work being done with her father, right?"*[I-604]

“*So we can monitor care because sometimes there are people we don't lose contact with and the progress of, but there are others that we don't know about the continuity of care after discharge... The care sharing could then allow us to monitor the person with dementia status here after discharge.”* [F-56]

Finally, the use of messages (in an integrated way) in a technological solution attracted the interest of both informal and formal caregivers, but was most welcomed by the formal ones. The most important requirement regarding the use of messaging, particularly between the two types of caregivers but also among formal caregivers, is to ensure that there is the possibility of private messaging:

“*Sometimes it might be important to have an exchange of ideas just between two professionals about the person that would not involve others. And if that was kept as a record for that person, it means that it would always be accessible there for later on, not forcing caregivers to have to be on their phone or Facebook to share that information just between themselves.”* [F-47]

Those design implications confirm the need for a coordinated, person-centered dementia care, further highlighting the value of structured personal profiles to support continuity of care, particularly when caregiving responsibilities are distributed.

### 4.5 Privacy and security

As we have seen, most informal caregivers are older than 50 years old, and people with dementia are usually older than 70 years old. Older people might not be familiar with new technologies and may be wary of technological solutions, particularly those in which they have to share their personal information.

Security and privacy concerns are still in place as, in therapy sessions, assistive technologies collect a great amount of sensitive information that should only be accessed by authorized personnel. Thus, the privacy of the information exchanged is very important, especially when other people are involved. Beyond ethical issues, failure to ensure the security and privacy of such information can prevent older people from accepting the assistive solutions developed. As such, we also addressed how comfortable caregivers would be inserting personal content on assistive technological solutions (assuming that only authorized people could consult the data). Informal and formal caregivers indicated they were comfortable introducing personal content into a technological system as long as it guarantees privacy and security.

Concerns about privacy, security, and technology adoption are well established in the literature. Previous studies have pointed out how ethical issues related to privacy and usability influence the acceptance of assistive technologies among people with dementia and their caregivers (92). Our design implications confirm these concerns and extend them, showing that both informal and formal caregivers are willing to use assistive technologies, even to share sensitive content, if, and only if, strict privacy guarantees are in place. This reinforces existing calls for ethics-based design principles and adds further evidence that privacy guarantees are not only regulatory or ethical requirements but also key factors for technology adoption in dementia care contexts, whether at home or in clinical settings.

### 4.6 Functional requirements and expected outcomes

**Figure 2:**
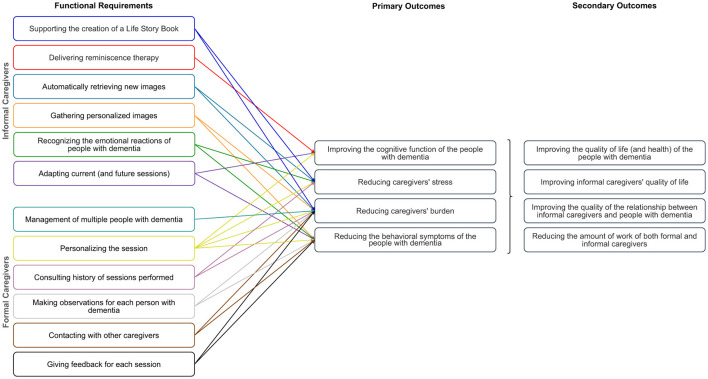
List of the proposed functional requirements for novel assistive technological solutions to support reminiscence therapy gathered for both formal and informal caregivers, as well as the corresponding expected primary and secondary outcomes (figure best seen in color).

In Figure 2, we present our proposed list of functional requirements for both formal and informal caregivers to be fulfilled by novel assistive solutions to support the creation of diversified, personalized, and emotion-aware reminiscence therapy sessions. The expected primary and secondary outcomes with the fulfillment of each requirement are also outlined.

Our list of requirements initially focused only on aspects related to reminiscence therapy regarding informal caregivers (based on the first survey). Later on, it was updated to include formal caregivers after applying the remaining surveys, interviews, and focus groups. The functional requirements presented in the manuscript reflect the full set identified through our user-centered design process. The criteria used to identify the relevant requirements were: (i) alignment with caregivers' needs, identified iteratively, based on the surveys, interviews, and focus groups; (ii) supporting at least one of the primary outcomes identified together with formal caregivers on the interviews and focus groups; (iii) technical and contextual feasibility within the intended use environment, with a particular focus on the household; and finally, (iv) helping to reduce caregivers' workload and stress. Note that some of the requirements outlined for one type of caregiver may also be useful for the other.

The expected primary and secondary outcomes were identified using the following procedure. First, we conducted a comprehensive review of existing interventions and frameworks targeting dementia care to identify outcomes commonly associated with cognitive support, caregiver burden, and quality of life improvements. Following, we used the follow-up interviews and focus group with clinicians and dementia care specialists to validate the outcomes and prioritize them based on real-world relevance and impact, i.e., the classification of outcomes into primary and secondary categories was made based on their perceived importance and impact. Each identified outcome was systematically mapped to specific system requirements using a goal-oriented design approach (as depicted in Figure 2). This ensured that each requirement addressed at least one primary outcome, reinforcing traceability. Below, we outline the anticipated benefits arising from the implementation of the identified functional requirements, highlighting their potential to improve the quality of reminiscence therapy and reduce caregiver burden.

One-to-one traditional reminiscence sessions can be a burden for caregivers, thus challenging to sustain over time in real-world settings (22, 32, 48, 70, 73). One reason for this is the pressure upon caregivers to nurture and sustain conversations with people with dementia: many questions are made by caregivers during sessions to which people with dementia passively respond. Delivering personalized and diversified reminiscence therapy with the help of assistive technologies can bypass the working memory difficulties that usually hinder conversations for people with dementia, leading to more engaging dialogue and an overall interesting and stimulating experience for all participants. If assistive technologies can automatically retrieve new and personalized multimedia for each person with dementia, the pool of multimedia available will increase without additional efforts from the caregivers. With this, we anticipate that the level of work and stress of the caregivers will reduce while the quality of the sessions will likely increase.

Creating life story books for each person with dementia may prevent the abandonment of therapy due to a lack of material to be used during sessions. It may also help caregivers to learn more about each person with dementia, which might be helpful in the course of the therapy, as well as to see the person with dementia in a new light. We believe this will help to sustain a better relationship between people with dementia and their caregivers by increasing their mutuality (i.e., their level of “closeness”) and social connectedness.

With assistive technologies able to fulfill most of the functional requirements proposed, people with dementia will be cognitively, sensorimotor-related, and verbally activated. This will be done by tailoring the therapy to the current condition of each person with dementia through a centered care where all the information available (biographical and emotional information, feedback collected from sessions, observations made, input from different caregivers) is taken into account to conduct and adapt the therapy sessions over time. We expect this might lead to an improvement of the current cognitive function of people with dementia (e.g., comprehension, language, memory, orientation, social behavior, and motivation) while reducing some of the behavioral symptoms (e.g., agitation, wandering) they show due to inactivity, discomfort and the need for social contact. It is further anticipated that intrapersonal benefits may also arise, namely, higher levels of engagement, sense of identity, self-awareness, personal stability, and life meaning.

Another important aspect is to ensure that the emotions experienced during therapy by people with dementia are taken into consideration, thus protecting (informal) caregivers from negative emotional reactions that may occur. This is particularly important given that each person has their memories associated with different stages of the disease. Ultimately, this could lead to unpredictable results, potentially eliciting negative feelings or unpleasant memories that may trigger aggressiveness or irritability in the person with dementia (7). On the one hand, the use of personalized and diversified material may be able to help regulate emotional fluctuations experienced by people with dementia. On the other hand, a system that can identify how a person with dementia feels toward a given stimulus would provide valuable clues for (informal) caregivers, thus allowing them to manage what content they want to use in the current (and future) session.

Allowing caregivers to provide (and consult) feedback/observations for each person with dementia will also be very valuable. Such observations might be seen as a diary of the person with dementia through the lens of the caregivers, which might be useful for future reminiscence therapy sessions, but for other therapeutics as well. All the information collected (from the sessions performed) will help caregivers to compare sessions and improve them over time. The feedback collected will also help understand the overall emotional state of the person with dementia alongside their reactions to the stimuli presented. Ultimately, it will help reduce caregivers' burden while reducing the person with dementia's behavioral symptoms. Finally, the possibility to easily contact other caregivers with whom a given caregiver shares the care of a person with dementia will provide centered care and reduce caregivers' burden.

In summary, with the fulfillment of most of the requirements outlined, the expected primary outcomes are the: improvement of the cognitive function of the people with dementia, reduction of caregivers' stress and caregivers' burden, and reduction of the behavioral symptoms of the people with dementia. As secondary outcomes, we foresee an overall improvement in the quality of life (and health) of the people with dementia and their caregivers, an improvement in the quality of their relationship, and a decrease in the amount of work caregivers will have in preparing and conducting the therapy.

## 5 *KeepsakeBox* prototype

In this section, we present the resulting architecture of the *KeepsakeBox* prototype and the preliminary feasibility study performed to assess its current version's usability and ease of use.

### 5.1 Proposed architecture

**Figure 3:**
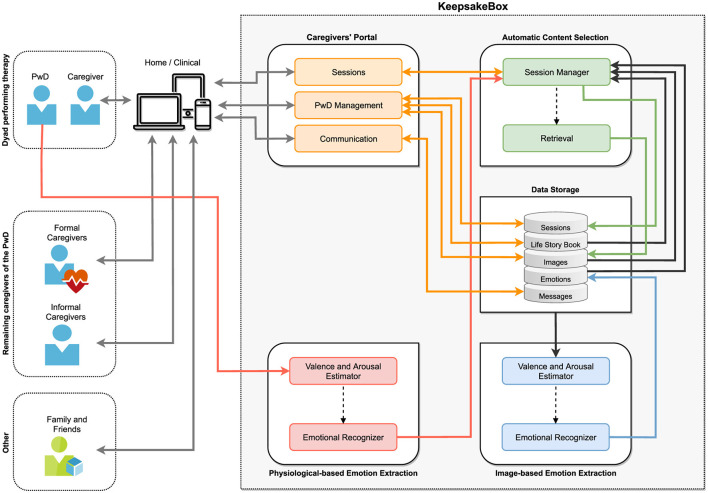
Main stakeholders of the reminiscence therapy ecosystem, and an overview of the architecture of the *KeepsakeBox* prototype. The solid arrows represent direct data flows between modules, while the dashed arrows indicate conditional, inferred, or feedback-based operations (figure best seen in color). PwD, Person with Dementia.

Figure 3 depicts the architecture of the *KeepsakeBox* and its ecosystem. On the left, we present the multiple stakeholders directly or indirectly involved in the reminiscence therapy: the people with dementia, their informal primary caregiver (usually the spouse or child), and/or a formal primary caregiver (neuropsychologist, geriatrician, psychiatrist, or clinician). The latter usually prescribe the therapy to be performed at home by the informal caregiver, but they may also carry it out in a clinical environment. Other caregivers, especially formal ones, who do not carry out therapy sessions but accompany people with dementia, may benefit from consulting the progress of the therapy sessions. Finally, other family members and close friends who compose the support network of the people with dementia (and the primary caregiver) can help provide information and multimedia content for the sessions. On the right, we present the architecture of *KeepsakeBox*. It was designed to allow, in an easy and friendly way, the various stakeholders to participate in the care of people with dementia. Following, we briefly describe the core ideas of each module.

#### 5.1.1 Caregivers' portal

This module allows caregivers to manage people with dementia, communicate with other caregivers, introduce biographical information about each person with dementia alongside images (to be used in therapy), create, personalize, and conduct the sessions, as well as consult the information collected from sessions.

**Figure 4:**
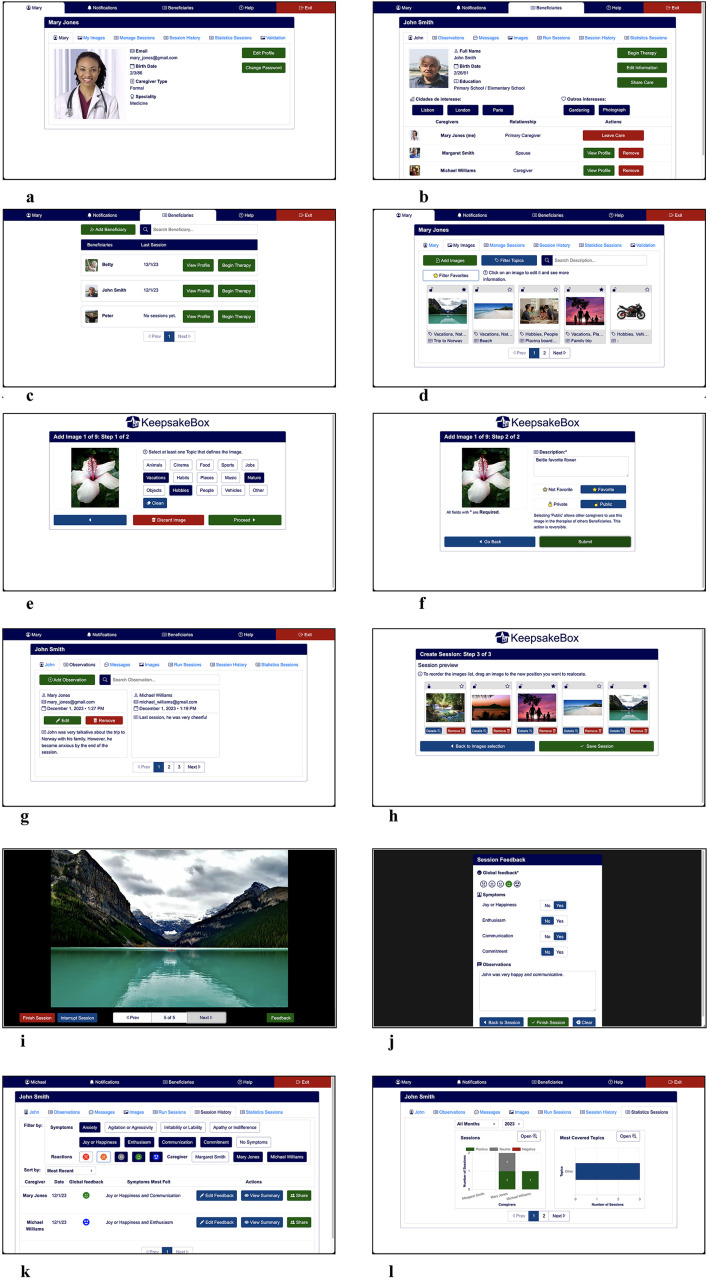
Examples of *KeepsakeBox* pages for the functionalities currently supported. **(a)** Caregiver's profile. **(b)** Person with dementia's profile. **(c)** List of people with dementia. **(d)** Images gallery. **(e)** Uploading image (step 1). **(f)** Uploading image (step 2). **(g)** Observations. **(h)** Creation of sessions. **(i)** Conducting a session. **(j)** Collecting feedback. **(k)** Consulting sessions' history. **(l)** Visualizing statistics (figure best seen in color).

As people age, physiological and cognitive changes are almost inevitable and must be compensated for (e.g., visual impairments). This is particularly relevant for informal caregivers since most are older. In the design of the Caregivers' Portal, we followed the Kurniawan et al. Web Design guidelines for older people (96), the recommendations from Piper et al. (69) and Freeman et al. (97), as well as the age-related differences in performance and aesthetic perceptions identified by Urbano et al. (98). Figure 4 depicts examples of the *KeepsakeBox* Caregivers' Portal pages for the functionalities currently supported. The list of implemented functionalities and corresponding descriptions is available as Supplementary material.

#### 5.1.2 Data storage

All the information regarding caregivers, people with dementia, sessions conducted, images, emotional information associated with each image/session, and all messages exchanged between caregivers are stored in this module (see Table 4). This data was validated through system-level consistency checks and confirmation with caregivers during interviews, focus groups, and the feasibility study.

**Table 4:** Information stored in the Data Storage module of the *KeepsakeBox* regarding caregivers, people with dementia, images, sessions, collected feedback, chat, and observations.

**Caregiver**	
	Name
	E-mail
	Mobile number
	Date of birth
	Type (informal or formal)
	Speciality (if formal) or relationship (if informal)
**Image**	
	Caregiver or person with dementia
	Categories (e.g., nature, people, places)
	Description
	If is personal
	If is private
	Image path
	Negative intensity
	Neutral intensity
	Positive intensity
**Session/image feedback**	
	Session
	Image
	Global emotional reaction
	Caregiver
	Date of feedback
	If the person with dementia was anxious
	If the person with dementia was aggressive
	If the person with dementia was angry
	If the person with dementia was apathetic
	If the person with dementia was happy
	If the person with dementia was enthusiastic
	If the person with dementia was communicative
	If the person with dementia was engaged
**Person with dementia**	
	Name
	Short name to be displayed
	Date of birth
	Education level
	Interests
	Cities (visited or lived in)
**Session**	
	Caregiver
	Person with dementia
	Start session date
	End session date
	If session is finished
	Duration
	Number of images to be shown
	Current image being shown
	Global emotional reaction
**Person with dementia chat**	
	Caregiver
	Person with dementia
	Date of last message read
	Date of last message send
	Date of message created
	Message
**Observation**	
	Caregiver
	Person with dementia
	Date of last update
	Observation

#### 5.1.3 Physiological-based emotion extraction

At the beginning of each session and during the visualization of the images, this module will collect and analyze the person with dementia's physiological signals to correctly identify their current emotional state. Negative reactions should be reported to the caregiver to adjust the course of the session if desired. This module is currently under development, leveraging the previous work of our research team (99–101).

Even though using sensors to collect physiological signals is promising, it has some limitations that need to be properly addressed. First, their use is still underexplored in aging populations, since existing AI models often fail to include sensor data from older adults or those with cognitive impairments. Second, the most reliable and accurate devices are costly and/or cumbersome to use. To deal with concerns about the comfort and usability of the sensors, we are focusing on non-intrusive, lightweight wearable devices. In particular, we are exploring the use of wearables like the Empatica E4 and Garmin Venu. These are designed to be discreet and easy to use, minimizing potential discomfort or confusion for individuals with cognitive impairments while enabling reliable physiological data collection.

Ethical considerations regarding data collection will be addressed through iterative testing, consultation with clinical partners, and adherence to established ethical guidelines.

#### 5.1.4 Image-based emotion extraction

Every time a new image is added to the solution, this module will analyze it to infer the emotional information it conveys and store that information in the corresponding database of the Data Storage module. This information will then be used by the Automatic Content Selection module to adjust the course of the therapy and during the creation of new sessions. This module is currently in the final stages of development. It leverages pretrained computer vision models capable of assessing emotional information conveyed by images (using publicly available affective image datasets for model calibration and evaluation) (102–104). Our goal is to semi-automatically estimate whether an image is likely to evoke positive, neutral, or negative emotions to support personalized and emotionally safe reminiscence sessions while allowing caregiver overrides to ensure flexibility and safety in content curation. Nonetheless, it is currently limited to the image domain, but we expect to include other media in the near future.

#### 5.1.5 Automatic content selection

This module will work as the bonding glue that connects all the modules to take advantage of all the information available to assist in creating personalized and diversified sessions. This module will be responsible for efficiently obtaining new images (when needed) related to the person with dementia's life and adapting the course of a session if strong negative emotional reactions occur. This is a particularly important issue because, during sessions, people with dementia often become agitated, aggressive, or angry, and caregivers do not know how to adequately deal with such situations (in particular, the informal ones).

### 5.2 Preliminary feasibility evaluation

In this section, we present the preliminary feasibility evaluation performed on the current version of the *KeepsakeBox* prototype through semi-structured interviews and focus groups. Our focus was on the usefulness of the currently available features and the prototype's overall usability.

#### 5.2.1 Participants

Two informal and 24 formal caregivers participated in the interviews and focus groups. One of the informal caregivers cares for her spouse, and the second one for her mother. Both caregivers were female.

The formal caregivers who participated in our semi-structured interviews and focus groups work in the dementia field at Alzheimer Portugal,^5^ Centro de Apoio Alzheimer Viseu,^6^ Hospital de Santa Maria,^7^ Irmãs hospitaleiras - Casa de Saúde de Idanha,^8^ and Santa Casa da Misericórdia de Lisboa.^9^ They were mostly women (22 out of 24) working as neurologists, neuropsychologists, nurses, occupational therapists, physical therapists, and social workers. They were all Portuguese.

#### 5.2.2 Procedure

Each remote session began with a brief explanation of what would be done in the testing session, the estimated session duration, and the objectives of the tests. Next, each caregiver was provided the Informed Consent Form to read and consent. If they agreed, they were invited to complete a questionnaire with their demographic information.

The research team provided a brief explanation of the *KeepsakeBox* prototype. The caregiver was then invited to test the different functionalities of the prototype using the think-aloud technique (to comment on their experience while using the prototype). We prepared the following task scenarios to guide caregivers to use all the functionalities available:

- *T*_1_: Register your account to start using this new platform;
- *T*_2_: You are currently taking care of a person with dementia who is undergoing reminiscence therapy (Mr. John Smith). Add him to the *KeepsakeBox*;
- *T*_3_: Someone else is caring for Mr. John. Give them access so that he/she can help in the care of Mr. John;
- *T*_4_: Once the other caregiver has accepted your request, send him/her a message asking if he/she can do a therapy session next week as you will be away;
- *T*_5_: During the day, you noticed that Mr. John was in a very good mood and chatty. Make an observation about that;
- *T*_6_: The pool of photos of Mr. John that are available to use in the *KeepsakeBox* is very small, so you decide to add some photos you have in a folder;
- *T*_7_: The other day, you found a set of interesting photos to use in the therapy. Add these pictures so that you can use them with the various people with dementia you care for;
- *T*_8_: Recently, you have not had much time to create specific sessions for each person with dementia you care for. So, you decide to create one session that can be used for several people. The session should contain four images about food, three about animals, and three about nature. When you look at the result, you find that the third picture in the session might make people sad. Replace that picture with a more cheerful one;
- *T*_9_: As your colleague Mary has been a little short of time to create new sessions, you decide to share this session with her so that she can use it;
- *T*_10_: Associate this session with Mr. John Smith so that in the future, you or another caregiver of Mr. John can conduct this session with him;
- *T*_11_: As you have some time this week, you decide to create a session tailored to Mr. John, with five images related to vacations, six with hobbies, and three with people. Sort the 14 images considering your purposes for the session;
- *T*_12_: It is the day to carry out a therapy session with Mr. John. Since he is in a good mood, you use the session you created previously with the 14 photos. Start the session and record Mr. John's reactions to each image (if you think it is necessary). After viewing five images, you have to end the session because Mr. John becomes sad and agitated. Record his overall reaction to the session.
- *T*_13_: You will conduct a new session with Mr. John. However, before you do, you want to see which images were used in one of the sessions in which he had no symptoms/reactions to understand which images affected him the least;
- *T*_14_: Since you will have some time today to create a session, you would like to use images of topics Mr. John likes. Check which topics this month have produced the most positive results;
- *T*_15_: You were on vacation last month and can no longer remember how many sessions you held in the previous month. Check how many negative sessions you had that month and with whom;
- *T*_16_: Starting next week, you can no longer care for Mr. John Smith. Stop being his caregiver and transfer his primary care to another caregiver.

After finishing all tasks, each participant was invited to fill out a questionnaire. This questionnaire included the System Usability Scale (SUS) (105) to evaluate the usability of the *KeepsakeBox*, and the first six questions from Technology Acceptance Model (TAM) (106, 107) to evaluate its perceived usefulness for the caregivers. The SUS consists of 10 statements to which users rate their levels of agreement regarding usability (using a Likert scale from 1 to 5), where half the statements are worded positively (even statements) and half are worded negatively (odd statements). TAM comprises 12 items where the first six assess perceived usefulness (PU) and the remaining six assess perceived ease of use (PEU). For each item, users rate their levels of agreement using a Likert scale from 1 to 7.

#### 5.2.3 Results

The evaluation conducted showed promising results. We achieved an average SUS score of 85 (grade A^+^), suggesting caregivers found *KeepsakeBox* easy to use and learn and would recommend it to other caregivers. These results are higher than those reported in the literature for healthcare applications (not necessarily related to technologies for dementia), which have a mean SUS score of 71.30 (standard deviation of 12.72) (108). Regarding the reliability of our results, internal-consistency coefficients showed a Cronbach's α of 0.84 for the even statements and 0.68 for the odd ones.

**Figure 5:**
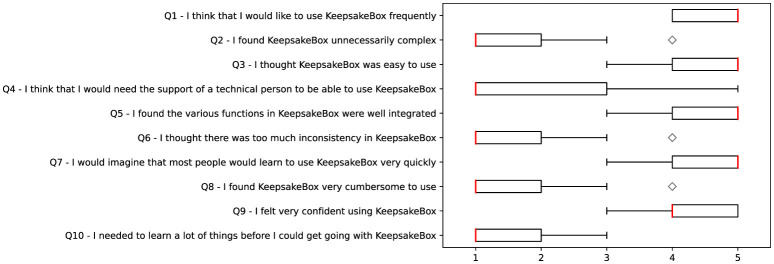
Boxplot showing the SUS score for each question (where 1 is strongly disagree and 5 is strongly agree). The diamond-shaped boxes indicate the presence of outliers.

**Figure 6:**
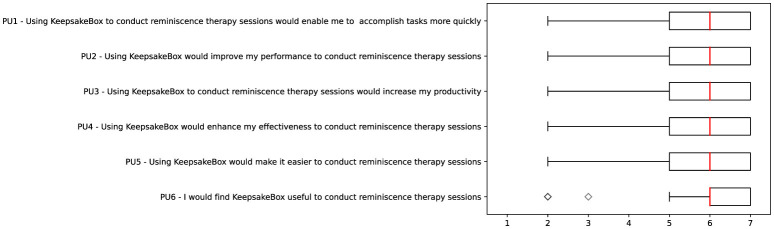
Boxplot showing the TAM score for each question regarding assessing the perceived usefulness (where 1 is extremely disagree and 7 is extremely agree). The diamond-shaped boxes indicate the presence of outliers.

The results of TAM regarding perceived usefulness suggest that caregivers think *KeepsakeBox* would help them to manage and conduct reminiscence therapy sessions more easily. The internal-consistency coefficients of our results' reliability showed a Cronbach's α of 0.99. Figures 5, 6 present, respectively, a boxplot for each question summarizing the score assigned by caregivers using SUS and TAM.

Overall, all the features presented were very well received by caregivers, the overall design of the platform was highly praised, and caregivers also showed their appreciation for being included in the design and evaluation of *KeepsakeBox*:

“*We are working with people who may have visual difficulty as well, so the font size and typeface is very important, and this one seems appropriate.”* [F-1]

“*I think it's simple compared to other applications. I think it's straightforward. Everything is simple and intuitive. The feeling I have is that I would use it with people.”* [F-5]

“*It's interesting, congratulations! I was pleasantly surprised. I look forward to further progress, and I hope to receive news soon (about new features).”* [F-52]

“*It is very good that in creating this platform, you are actually getting information and evaluating it with the people who are going to use them.”* [F-57]

“*As a technician, I would like to thank you for your willingness to listen to the technicians. Because many times these tools come to us as a final product, and we are not given this possibility. Thank you for actually involving us in such an active way. We really feel that it's going to be a valuable asset. It will be a product that will be transversal to several specialties, and we can already see its potential at this stage.”* [F-62]

## 6 Conclusions, limitations, and future work

Assistive technologies centered on the real needs of people with dementia's caregivers may play an important role in dementia care. To foster the design of such assistive technologies, we presented a user-centered study composed of worldwide cross-sectional surveys, follow-up semi-structured interviews, and focus groups. Seven hundred and thirteen informal and 67 formal caregivers of people with dementia participated in the study.

Our findings revealed that novel assistive solutions must provide mechanisms to carry out the therapy in a simple way, as well as diversify and personalize the current session (and following ones) based on both the biographical information of the people with dementia and their emotional reactions (since people with dementia often become agitated, aggressive or angry, and informal caregivers do not know how to deal with it). Formal caregivers need an easy way to manage multiple people with dementia and communicate with other caregivers, mechanisms to create therapy sessions, collect feedback after conducting each session, and consult the history of sessions performed (in particular, to identify images that triggered negative emotional reactions, and consult any notes taken about them).

Thanks to the valuable time and experience of the informal and formal caregivers who participated in our study, we closed this work with a set of validated functional requirements gathered for both formal and informal caregivers, as well as the expected primary and secondary outcomes with the fulfillment of each requirement. We also presented the architecture of the *KeepsakeBox* prototype developed during the requirements elicitation process. This architecture can be used as a basis to develop forthcoming technological solutions for reminiscence therapy. It was designed to accommodate all the identified requirements and support the participation of all the stakeholders involved in the care of people with dementia. Our preliminary evaluation of the *KeepsakeBox* prototype showed promising results using the SUS and TAM questionnaires. Caregivers found *KeepsakeBox* easy to use and learn, and a valuable tool to help them manage and conduct reminiscence therapy more easily.

We acknowledge that this work has the following limitations that warrant discussion and will also shape our future work. Although we have followed a user-centered approach, focusing on the needs of formal and informal caregivers as the primary users of assistive technologies such as *KeepsakeBox*, we recognize that we have not directly investigated the needs, preferences, or experiences of people with dementia. Our decision was based upon the fact that caregivers are the ones who interact with the system and make decisions about the content and delivery of the sessions.

Our study presents early-stage feasibility results based on the preliminary evaluation of the *KeepsakeBox* prototype with 26 participants, including 24 formal caregivers and two informal caregivers. We did not involve people with dementia as participants, since they are not the primary users of the application. The inclusion of formal caregivers allowed us to gather expert insights into dementia care practices and evaluate our prototype's use in realistic clinical settings. However, we acknowledge the underrepresentation of informal caregivers in this evaluation. They are the primary users of assistive technologies at home; therefore, their perspectives are important for assessing long-term usability. Thus, our results should be interpreted as preliminary and mainly indicative of feasibility and usability, rather than conclusive evidence of effectiveness or generalization. Moreover, our results are a good indicator, but they should be considered with caution since it is known that there may be a positive bias on the part of the participants.

To better evaluate these aspects, and after finishing the implementation of all the modules proposed, our future work is to conduct an in-the-wild evaluation over a large period of time with a more balanced sample that includes a broader and more diverse population of formal and informal caregivers, as well as people with dementia, to better reflect real-world usage scenarios. As a result, we will be able to provide a ready-to-use assistive solution, *KeepsakeBox*, which can be used both at home and/or in institutional environments.

## Data Availability

The raw data supporting the conclusions of this article will be made available by the authors, without undue reservation.

## Footnotes

^1^A Keepsake box is a memento or memory box, usually made of wood, which is used to store memories of a special moment, event, or person, often with emotional value.

^2^ https://facebook.com/

^3^ https://docs.google.com/forms/

^4^ https://zoom.us/

^5^ https://alzheimerportugal.org/

^6^ https://obrassociaisviseu.pt/centro-de-apoio-alzheimer/

^7^https://chln.min-saude.pt/.

^8^ https://irmashospitaleiras.pt/unidade-de-idanha/

^9^ https://scml.pt/
