# Supplementary material for: Developing assistive technology to support reminiscence therapy: a user-centered study
Source: Front Med (Lausanne). 2025 Oct 2;12:1625897. doi: 10.3389/fmed.2025.1625897 (PMC12529597; doi:10.3389/fmed.2025.1625897)
Supplement: Supplementary file 1 [file Table_1.pdf]

Supplementary material

The current version of the KeepsakeBox comprises the following functionalities:

- **Register and Login** the caregiver account to use *KeepsakeBox*;
- **Managing the Profile of the Caregiver**, with the caregiver's information, such as name, email, mobile number (optional), type of caregiver (formal or informal), medical specialty (if formal), relationship with the person with dementia (if informal), and a profile picture (optional). The email is used as the caregiver's unique identifier (see Figure 4a);
- **Managing the Profile of the Person with Dementia**, with biographic information, such as name, date of birth, education, name to be presented, a profile picture (optional), and the list of caregivers associated with their care (see Figure 4b);
- **Listing the People with Dementia**, which allows caregivers to see a list of all the people with dementia they care for, and search by their names (see Figure 4c);
- **Consulting Caregivers' Images Gallery**, which is similar to the P**erson with Dementia's Images Gallery**, but the caregiver consults their own list of images (that can be used with all the people with dementia they care for). Searching, filtering, and editing are also available (see Figure 4d);
- **Uploading** multiple images (or a folder). Each image could have an accompanying description, a set of categories that describe it, can be starred as a favorite, and can be set as public or private (see Figures 4e and 4f). This information is useful to create a life story book of the person with dementia to be used as a reminiscence tool to support recollecting autobiographical memories;
- **Consulting the Person with Dementia's Images Gallery**, which shows the list of images (and accompanying information) of a given person with dementia. It is possible to edit and filter/search images to be shown by category, description, and favorites;
- **Messaging**, which allows caregivers to communicate with other (registered) caregivers of the person with dementia;
- **Sharing the Person with Dementia's Care**, which allows the primary caregiver to share the care of a given person with dementia with other (registered) caregivers. This is important for both informal caregivers (to feel more supported, less burdened, and less isolated) and formal ones (to optimize the relationship with other caregivers and improve the care provided). The invited caregiver must accept the invitation to participate in the care;
- **Managing Observations**, which allows caregivers to add new observations and consult/edit the existing ones. They might be seen as a diary of the person with dementia through the lens of the caregivers, which might be useful for future reminiscence therapy sessions, but for other therapeutics as well (see Figure 4g);
- **Notifications** of new messages, observations, and invitations to participate in the care of a person with dementia;
- **Creation of Sessions** for reminiscence, where a set of images is selected according to the desired specifications, for example, considering the topics to be addressed in the session. This choice can be done manually (by the caregiver), semi-automatically, or fully automatically (by the *KeepsakeBox* prototype). The sessions created can be shared between caregivers to use with multiple people with dementia (see Figure 4h);
- **Conducting a Session**, by displaying the selected set of images (see Figure 4i);
- **Collecting Feedback** during sessions regarding the emotional reactions of the person with dementia to each image and the session as a whole (see Figure 4j);
- **Consulting Sessions' History** to view the details of each session held to help caregivers review all the information collected in an easy and simple way (see Figure 4k);
- **Visualizing Statistics** regarding the sessions performed, allowing caregivers to view the number of sessions that triggered positive, neutral, or negative feelings and the themes that were addressed more often. This information is essential for the preparation and delivery of future sessions (see Figure 4l).
